# Molecular phylogeny of human adenovirus type 41 lineages

**DOI:** 10.1093/ve/veac098

**Published:** 2022-10-20

**Authors:** Jasper Götting, Anne K Cordes, Lars Steinbrück, Albert Heim

**Affiliations:** Institute of Virology, Hannover Medical School, Carl-Neuberg-Str. 1, Hannover 30625, Germany; Institute of Virology, Hannover Medical School, Carl-Neuberg-Str. 1, Hannover 30625, Germany; Institute of Virology, Hannover Medical School, Carl-Neuberg-Str. 1, Hannover 30625, Germany; Institute of Virology, Hannover Medical School, Carl-Neuberg-Str. 1, Hannover 30625, Germany

**Keywords:** phylogeny, adenoviruses, hepatitis, evolution, recombination, F41

## Abstract

Type 41 of human adenovirus species F (HAdV-F41) is a frequent aetiology of gastroenteritis in children, and nosocomial as well as kindergarten outbreaks have been frequently described. In contrast to other HAdV types, HAdV-F41 was not associated with a life-threatening disseminated disease in allogeneic haematopoietic stem cell transplant (HSCT) recipients or any severe organ infections so far. Due to the limited clinical significance, the evolution of HAdV-F41 has not been studied in detail. Recently, HAdV-F41 has been associated with severe hepatitis in young children, and interest in HAdV-F41 has skyrocketed, although the aetiology of hepatitis has not been resolved. Complete genomic HAdV-F41 sequences from thirty-two diagnostic specimens of the past 11 years (2011–22) were generated, all originating from gastroenteritis patients. Additionally, thirty-three complete HAdV-F41 genomes from GenBank were added to our phylogenetic analysis. Phylogenetic analysis of sixty-five genomes indicated that HAdV-F41 evolved with three lineages co-circulating. Lineage 1 included the prototype ‘Tak’ from 1973 and six isolates from 2007 to 2017 with an average nucleotide identity of 99.3 per cent. Lineage 2 included 53 isolates from 2000 to 2022, had an average nucleotide identity of 99.8 per cent, and split into two sublineages. Lineage 3, probably described for the first time in 2009, had a 45-nucleotide deletion in the long fibre gene and had evolved significantly in the short fibre and E3 region. Moreover, a recent Lineage 3 isolate from 2022 had a recombinant phylogeny of the short fibre gene. Fibres interact with cellular receptors and determine cellular tropism, whereas E3 gene products interfere with the immune recognition of HAdV-infected cells. This in-depth study on the phylogeny of HAdV-F41 discovered significant evolution of recently described Lineage 3 of HAdV-F41, possibly resulting in altered cellular tropism, virulence, and pathophysiology.

## Introduction

Human adenoviruses (HAdVs) are non-enveloped, icosahedral DNA viruses, first isolated in 1953 from human adenoidal tissue (Rowe et al. 1953; Hilleman and Werner 1954) and belong to the *Mastadenovirus* genus. Their linear double-stranded DNA genome is ∼35 kb in length and encodes about 30–40 proteins (Davison, Benkő, and Harrach 2003). HAdVs are further separated into seven species (A–G) by phylogenetic criteria and subdivided into 113 types with type numbering merely related to their date of first isolation. HAdV types were initially defined by cross-neutralisation (HAdV serotypes 1–51) and later by complete genomic sequencing including the phylogenetic and recombination analysis of genes coding for the three major capsid proteins: penton, hexon, and fibre (genotypes 52–113) (Jones et al. 2007; Seto et al. 2011).

Only two types (40 and 41) are members of species HAdV-F. HAdV-F41, prototype strain ‘Tak’, was isolated in 1973 from the stool of a child suffering from gastroenteritis in the Netherlands (de Jong et al. 1983). HAdV-F41 is associated almost exclusively with gastroenteritis, most frequently in the toddler age, and is the second most frequent cause of diarrhoea in this age group, only second to Rotavirus (Lee, Damon, and Platts-Mills 2020). HAdV-F41 frequently causes nosocomial outbreaks in paediatric wards and community facilities such as kindergartens, but the disease remains limited to the gut even in severely immunosuppressed children although DNAaemias can be observed (Mattner et al. 2008; Gonçalves et al. 2011; Lefeuvre et al. 2021). Only a single case of severe HAdV-F41 dissemination has been reported in a stem cell transplant recipient (Slatter et al. 2005). This uniquely restricted organ tropism of HAdV-F41 can be attributed to the absence of an arginine-glycine-aspartic acid motif in its penton base, which binds types of all other HAdV species to its secondary cellular receptors, α_v_β_3_ and α_v_β_5_ integrins (Wickham et al. 1993; Albinsson and Kidd 1999). Recently, it was reported that the short fibre of HAdV-F41 binds to cells via heparan sulphate (Rajan et al. 2021), which may restrict its cellular tropism. In contrast, the long fibre of HAdV-F41 binds to the cellular coxsackievirus and adenovirus receptor as many other types of species HAdV-A, -C, -D, and -E do (Roelvink et al. 1998).

New types may evolve via diversifying selection (immune escape) of the neutralisation determinant (Robinson et al. 2013). Recombination between different types of the same HAdV species (‘intertypic recombination’) is also an essential mechanism for the evolution of species HAdV-B, -C, and -D types (Robinson et al. 2013). However, with its two types, HAdV-F hardly offers many options for intertypic recombination in contrast to species HAdV-D with its many types. Nevertheless, multiple distinct strains of HAdV-F41 have been distinguished by different growth characteristics, multiple restriction enzyme polymorphisms, and reactivity with different neutralising monoclonal antibodies (de Jong et al. 1983; van der Avoort et al. 1989). Despite these early studies, the molecular phylogeny of HAdV-F41 has not yet been studied in detail because of its limited clinical significance as a mere gastroenteritis virus. However, HAdV-F41 DNAaemia was recently associated with severe hepatitis in young children (Baker et al. 2022; Marsh et al. 2022).

Therefore, we sequenced the genomes and analysed the molecular phylogeny of 32 HAdV-F41 clinical isolates from 2011 to 2022, all originating from gastroenteritis cases. Moreover, thirty-three recently published complete genomic HAdV-F41 sequences from GenBank were included in the phylogenetic analysis.

## Materials and methods

### HAdV-F41 isolates and complete genomic sequences

HAdV-F41–positive samples (stool or cell culture supernatant from A549 cultures used for virus isolation) originating from the collection of the German National Reference Laboratory (Konsiliarlabor) for Adenoviruses were sequenced as described below. Furthermore, all thirty-three available complete genomic HAdV-F41 sequences from GenBank were included in the phylogenetic analysis. Sequences from the study by Tahmasebi et al. (2020) were excluded due to incompleteness and unusual number of single-nucleotide polymorphisms (SNPs).

### Ethical statement

The study only analysed viral data without patient material and thus did not require approval from the ethics committee.

### High-throughput sequencing and *de novo* assembly

DNA was extracted from 400 µl HAdV-F41–positive stool or cell culture supernatant (depending on the availability and virus load) using a Qiagen Blood Kit on a QIAcube. Library preparation was performed using the NEBNext Ultra II FS DNA Library Prep Kit for Illumina according to the manufacturer’s protocol. Final libraries were inspected on an Agilent Bioanalyzer, normalised, multiplexed, and sequenced on an Illumina MiSeq using a 600v3 Reagent Kit to generate 2 × 300 bp paired-end reads with an average of 1.25 million reads per sample.

*De novo* assembly was performed as previously described (Dhingra et al. 2019). Briefly, human reads were removed, and viral reads were trimmed with fastp and assembled with SPAdes, which usually resulted in a single high-coverage contig constituting the entire HAdV-F41 genome. Finally, genomes were polished using Pilon (Walker et al. 2014), and genome termini were manually examined and corrected using a mapping of the reads against the HAdV-F41 reference GenBank sequence (DQ315364). The resulting HAdV-F41 genomes were annotated from the HAdV-F41 reference sequence using Geneious Prime 2020.1.2. Finally, genomes were deposited in GenBank (accession numbers ON442312–ON442330, ON532817–ON532827).

### Phylogenetic analysis

Multiple sequence alignment of the complete sequences was carried out using MAFFT v7.450 (Katoh and Standley 2013). Phylogenetic trees were constructed with the HAdV-F40 reference sequence as the outgroup using RAxML v8 under the GTR GAMMA model with 500 rapid bootstrapping replicates and search for the best-scoring maximum likelihood (ML) tree (Stamatakis 2014). For comparison, the complete genomic sequence phylogeny was additionally inferred using MrBayes 3.2.6 (GTR & invgamma model with 500,000 MCMC steps and 50,000 burn-in steps) and Geneious Tree Builder (neighbour-joining with default parameters and 1000 bootstrap replicates) (Ronquist et al. 2012). The SimPlot 3.5.1 software was used to generate similarity plots (simplots) and to perform BootScan recombination analyses using default parameters, a window size of 1000 bp (BootScan) or 1,500 bp (simplot), and a step size of 200 bp (BootScan) or 300 bp (simplot) (Lole et al. 1999). TreeTime with default parameters was used to attempt inference of a time-calibrated ML phylogeny (Sagulenko, Puller, and Neher 2018). *In silico* restriction fragment length polymorphism (RFLP) analysis was performed in Geneious Prime 2020.1.2 (Biomatters) with the ten restriction enzymes used in the previous RFLP genome typing work (BamHI, BglI, BstEII, EcoRI, HindIII, KpnI, PstI, SacI, SmaI, and XhoI) (van der Avoort et al. 1989). Complete genomic sequences were digested with each enzyme separately, calculated fragments were rounded to the nearest 100 bp length, and fragments shorter than 400 bp were discarded to match the data with the fragment patterns from Fig. 1 of Johansson et al. 1991. A 137-bit binary string representing the presence or absence of all 137 occurring fragments from all enzymes was compiled for all complete genomic sequences as well as the twenty-four described genome types from Table 1 in the study by van der Avoort et al. (1989). All phylogenetic trees were visualised and annotated in R using ggplot2 and ggtree (Wickham 2016; Yu 2020).

**Figure 1.**
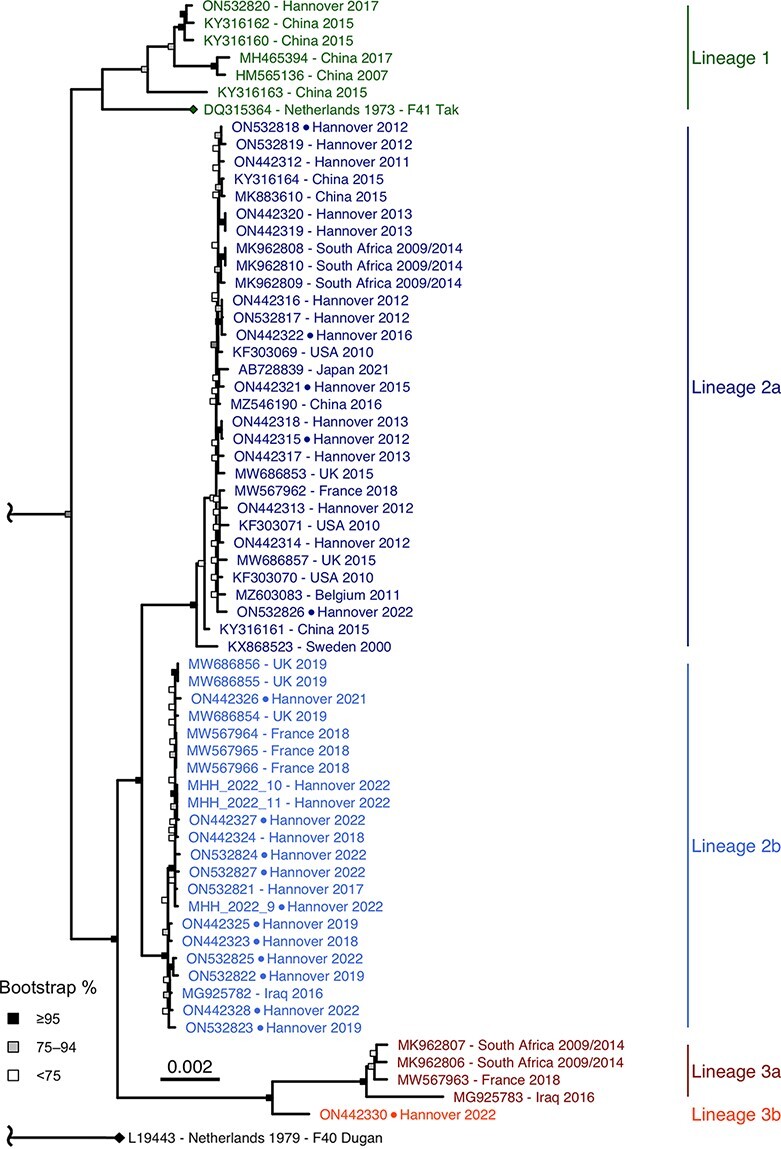
HAdV-F41 complete genomic sequence phylogeny. ML phylogeny of 65 HAdV-F41 genomes with the HAdV-F40 prototype as the outgroup. The total distance to the HAdV-F40 prototype was shortened due to the low genetic identity to the HAdV-F41 sequences (85.5 per cent identity with the F41 prototype). The two prototype sequences (HAdV-F41 DQ315364, HAdV-F40 L19443) are marked with a rhombus at the tip point. Sequences derived from cell culture-isolated virus are marked with a dot between accession number and sampling location. Bootstrap support values were binned into three categories (≥95 per cent, 75–94 per cent, and <75 per cent) indicated by filled boxes at the node points.

### Analysis for positive selection

Branch-Site Unrestricted Statistical Test for Episodic Diversification (BUSTED), as implemented on datamonkey.org, was utilised to identify genes under positive or diversifying selection (Weaver et al. 2018). All genes displaying high diversity in the SimPlot were analysed in BUSTED, which tests for gene-wide, non-site–specific selection (Murrell et al. 2015).

## Results

### HAdV-F41 phylogeny

Phylogenetic analysis of sixty-five complete genomic HAdV-F41 sequences exhibited three distinct lineages containing multiple identical or barely divergent sequences (up to 99.9 per cent identity within lineages) (Fig. 1). Clustering of lineages was stable between different tree models (ML, neighbour-joining, and Bayesian inference; see Supplementary Figs 1 and 2) and confirmed by bootstrapping.

Lineage 1 included the prototype ‘Tak’ from 1973 and isolates from as late as 2017. Only 7 of 65 complete genomic sequences were clustered as Lineage 1, but these originated from multiple geographic regions (China, the Netherlands, and Germany). Lineage 1 had a 99.3 per cent average nucleotide identity and even 99.2 per cent identity between the 1973 prototype and the last available isolate from 2017. Lineage 2, which had two sublineages (2a and 2b), included the majority of the genomic sequences (53 of 65), which originated from 2000 to 2022 and originated from multiple regions (Germany, Belgium, Japan, the USA, Sweden, China, South Africa, France, the UK, and Iraq). The nucleotide identity averaged 99.8 per cent within Lineage 2; 99.9 per cent within sublineage 2a and 99.9 per cent in sublineage 2b. The average identity between Lineages 1 and 2 was high (98.9 per cent), whereas Lineage 3 was more divergent from Lineage 1 (98.2 per cent). Lineage 3 included two sublineages, but sublineage 3b was represented only by a single sequence originating from Germany in 2022. Sublineage 3a included only four sequences originating from South Africa, France, and Iraq, during 2009–18. Nucleotide identity within Lineage 3 averaged 99.6 per cent.

Intratypic evolution was so slow that constructing time-calibrated complete genomic sequence phylogenies was unsuccessful (Supplementary Fig. S4).

### Evolution of genome regions

**Figure 2.**
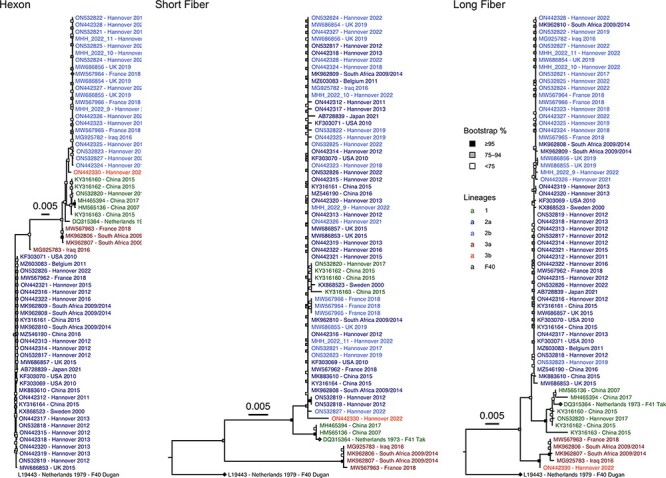
Phylogenetic trees of the hexon, short fibre, and long fibre genes of all 65 HAdV-F41 complete genomic sequences. The HAdV-F41 and -F40 prototype sequences are marked with a rhombus at the tip point. Bootstrap support values were binned into three categories (≥95 per cent, 75–94 per cent, and <75 per cent) indicated by filled boxes at the node points.  The total distance to the HAdV-F40 prototype was shortened in the hexon phylogeny due to the low genetic identity to the HAdV-F41 sequences (∼81 per cent).

Hotspots of evolution separating the lineages were found in the hexon, the long fibre, and the short fibre genes (Fig. 2), but the penton base gene was highly conserved (99.6–100 per cent, Supplementary Fig. S3). Only sublineage 2a has evolved significantly in the hexon gene with seven non-synonymous mutations in the loops of the neutralisation determinant ε. However, the BUSTED algorithm did not confirm the positive selection of potential immune escape variants (*P *= 0.087). Two Lineage 3 hexon sequences did not cluster with other Lineage 3 sequences, but this was not supported by significant bootstrap values. Lineage 3 had a 45-nucleotide deletion in the long fibre gene resulting in a 15-amino acid shorter fibre shaft. Furthermore, the long fibre gene of Lineage 3 had twenty-one SNPs (nine of these non-synonymous) compared to the consensus sequence. Four of nine amino acid substitutions were located in the fibre knob which binds the cellular receptor and haemagglutination-inhibiting antibodies. Evidence for positive selection was found by the BUSTED (*P *= 0.000) algorithm for the entire long fibre gene but not for the knob (*P *= 0.326). Only sublineage 3a has evolved significantly in the short fibre gene with 51 SNPs compared to the consensus sequence resulting in 20 amino acid substitutions, with four in the knob region. However, positive selection was not confirmed by the BUSTED algorithm (*P *= 0.444 for the entire short fibre gene, *P *= 0.5 for the short fibre knob).

**Figure 3.**
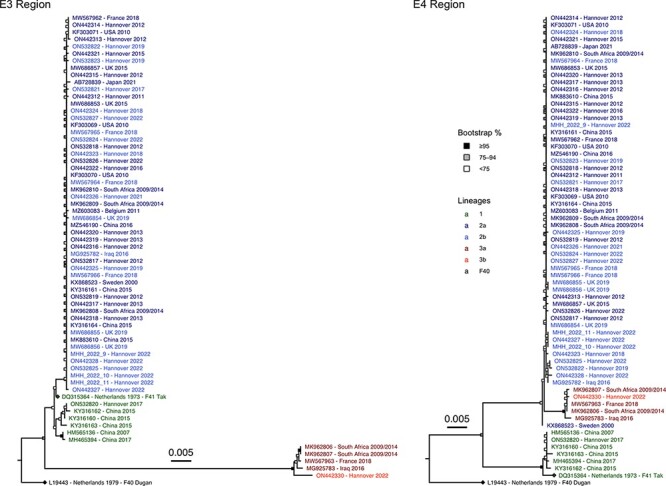
Phylogenetic trees of the E3 and E4 gene regions of all 65 HAdV-F41 complete genomic sequences. The HAdV-F41 and -F40 prototype sequences are marked with a rhombus at the tip point. Bootstrap support values were binned into three categories (≥95 per cent, 75–94 per cent, and <75 per cent) indicated by filled boxes at the node points.

Other hotspots of evolution were in the gene regions E3 and E4, coding for non-structural proteins (Fig. 3). Lineage 3 had the lowest average sequence identity (93.6 per cent) compared to the prototype sequence in the E3 region, even lower than the sequence identity between HAdV-F41 prototype ‘Tak’ and HAdV-F40 prototype ‘Dugan’ (98.3 per cent). Sixty-four of 156 SNPs were non-synonymous, but the BUSTED algorithm did not confirm positive selection in any of the five E3 open reading frames (ORFs) (*P *= 0.102 to *P *= 0.5). In the E4 region, Lineage 1 had sixty-two SNPs compared to the HAdV-F41 consensus sequence, with twenty-three being non-synonymous. Twenty-two of these were located in the E4 ORF4 and ORF6; however, no evidence for positive selection was found by BUSTED (*P *= 0.5).

### Interlineage recombination

**Figure 4.**
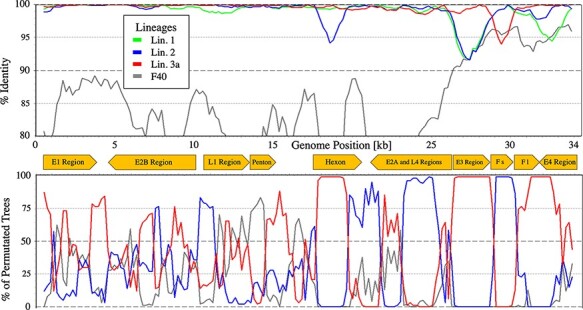
HAdV-F41 sublineage 3b BootScan. BootScan plot of the sublineage 3b genome with the consensus genomes of Lineages 1, 2, and 3a and HAdV-F40 as the outgroup.

Recombination between the two HAdV-F40 and HAdV-F41 (intertypic recombination) was not observed in the phylogeny of HAdV-F41 lineages. However, the short fibre gene region of sublineage 3b was phylogenetically linked to Lineage 2, and the recombinant origin of this region was confirmed by bootscanning (Fig. 4). The genome regions (E3, long fibre, and E4) around the short fibre were phylogenetically clearly related to sublineage 3a, while the genomes from the 5ʹ-end to the E3 region were too similar between Lineage 2 and sublineage 3a to show clear attribution in the BootScan.

Since no further interlineage recombinations were observed in the phylogeny of circulating HAdV-F41 strains, recombination was probably not a significant driver of the evolution of HAdV-F41 lineages.

## Discussion

HAdV-F41 is a highly prevalent gastroenteritis virus (Lee, Damon, and Platts-Mills 2020) which has been isolated as late as 1973 because it is fastidious (Kidd and Madeley 1981; de Jong et al. 1983). As HAdV-F41 is associated almost exclusively with gastroenteritis, interest in its evolution waned after an initial study applying RFLP had described several ‘DNA variants’ (van der Avoort et al. 1989).

Although HAdV-F41 has been highly endemic as a gastroenteritis pathogen for decades, no cases were found in our collection between September 2019 and December 2021, and the incidence was also reduced in England and Scotland probably due to hygiene measures implemented in Europe during the COVID-19 pandemic (Brauner et al. 2021; Marsh et al. 2022; UK Health Security Agency 2022a). Surprisingly, an association of HAdV-F41 with hepatitis in children was described in the UK and the USA during the resurgence of HAdV-F41 infections in late 2021 and early 2022 (Gutierrez Sanchez et al. 2022; Kelgeri et al. 2022), and circulation of a novel, highly pathogenic strain of HAdV-F41 could be suspected.

Therefore, we generated complete genomic sequences of re-emergent HAdV-F41 clinical isolates (from 2021 and 2022) and archival isolates from 2011 to 2019. All these originated from gastroenteritis cases as hepatitis cases were unavailable to us. Generated data could either detect a novel strain during re-emergence or be compared to partial HAdV sequences generated from recently described hepatitis patients.

The vast majority of our complete genomic sequences, as well as available GenBank sequences, were clustered as Lineage 2 (see Fig. 1). This lineage—together with Lineage 1 containing the prototype strain ‘Tak’ from 1973—probably represented the abundant gastroenteritis strains encountered worldwide over several decades. Sublineage 2a may represent a partial immune-escape variant due to its mutations in the neutralisation determinant ε. Lineage 3 is characterised by a 15-amino acid deletion of the 15th repeat in the long fibre shaft, somewhat similar to the deletion of the 14th repeat in the long fibre shaft of HAdV-F40 (Kidd, Erasmus, and Tiemessen 1990). Moreover, four amino acid substitutions were located in the long fibre knob, potentially affecting the binding to the cellular receptor. Furthermore, the E3 region of Lineage 3 was highly divergent from all other species HAdV-F sequences (HAdV-F40 and HAdV-F41 Lineages 1 and 2). Most E3 proteins exhibit immunomodulatory functions and can thus contribute to the virulence of an HAdV strain (Windheim, Hilgendorf, and Burgert 2004).

Only sublineage 3a was significantly divergent in the short fibre, with four amino acid substitutions in the knob region. As the short fibre knob of HAdV-F41 was recently reported to bind to heparan sulphate, potentially restricting the cellular tropism, these mutations in sublineage 3a might lead to a circumvention of this restriction (Rajan et al. 2021). Surprisingly, the short fibre gene of sublineage 3b was found to have a recombinant phylogeny derived from Lineage 2. Only a single sublineage 3b strain (from February 2022) was present in sixty-five complete genomic HAdV-F41 sequences; thus, sublineage 3b may be considered as a novel recombinant strain. However, *in silico* RFLP analysis of the only sublineage 3b sequence revealed a genome type D6 (see Supplementary Table S2), which was already isolated in 1980 in the Netherlands and had the fifteen-amino acid deletion in the long fibre shaft (van der Avoort et al. 1989; Kidd, Erasmus, and Tiemessen 1990). Identical RFLP patterns only elucidate the cutting sites of the ten used restriction enzymes and thus do not preclude significant divergence in other genome regions, which may have evolved recently and may influence tropism and virulence. Sublineage 3a genomes, on the other hand, were first identified by complete genomic sequencing of two samples from South Africa originating between 2009 and 2014 (MK962806 and MK962807). Furthermore, sublineage 3a genomes were found in Iraq (MG925783) and France (MW567963) as late as 2018 (Lefeuvre et al. 2021) but not in the present sequencing effort. The latter sequence originated from an HSCT patient and the detailed virus load data were published (patient B in Lefeuvre et al. 2021). Interestingly, virus loads in stool were rather low (about 10^6^ c/ml) compared to other patients and DNAaemia appeared rather late and not in parallel to the peak virus load in stool as in other patients. Suggestively, this is somewhat similar to the DNAaemia pattern in the recently described hepatitis cases in children (Baker et al. 2022; Gutierrez Sanchez et al. 2022; Kelgeri et al. 2022), but the highly immunocompromised patient B did not suffer from hepatitis. In spite of this similarity, the only available complete genomic HAdV-F41 sequence (GenBank #ON561778.1) originating from a paediatric hepatitis case clustered with sublineage 2b. Hepatitis cases in children had low, cell-associated virus loads (<10^5^ c/ml) in the peripheral blood (Gutierrez Sanchez et al. 2022; Kelgeri et al. 2022; Marsh et al. 2022), whereas typical adenovirus hepatitis causes high virus loads (usually >10^8^ c/ml) in the plasma (Ronan et al. 2014; Schaberg et al. 2017). Moreover, adenovirus hepatitis is associated with life-threatening disseminated disease in severely immunosuppressed patients, e.g. HSCT recipients (Forstmeyer et al. 2008; Lion 2014; Onda et al. 2021). However, these typical hepatitis cases were rather caused by HAdV types of species C, whereas HAdV-F41 disease remained limited to the gastrointestinal tract even in HSCT recipients in spite of mediocre virus loads in the blood (Mattner et al. 2008; Lefeuvre et al. 2021). In the recent cases of severe hepatitis in children, detection of adenovirus antigens or viral particles failed in explanted liver specimens (Baker et al. 2022; Gutierrez Sanchez et al. 2022), whereas in typical adenovirus hepatitis, these can be found in abundance (Forstmeyer et al. 2008; Onda et al. 2021).

Perhaps, an immune-mediated pathomechanism of liver injury, which is triggered by HAdV-F41 replication in other body sites (e.g. lymphoid tissue), can be suspected because severe hepatitis cases in children were associated with higher HAdV loads in the blood (UK Health Security Agency 2022a). Another speculative pathomechanism could be co-infection with adeno-associated virus 2 (AAV2), a dependoparvovirus, which may replicate in HAdV-F41–infected cells in other body sites than the liver. High levels of AAV2 DNA in the liver and AAV2 DNAaemia were found in hepatitis patients, in contrast to only low levels of HAdV-F41 DNA (Morfopoulou et al. 2022). AAV2 may perhaps cause an abortive infection of liver cells and thus liver cell injury or trigger an immunopathology against AAV2 antigens in the liver cells. A significant number of AAV2 reads was not found in the present study on HAdV-F41 from gastroenteritis patients.

In conclusion, three lineages of HAdV-F41 co-circulated for many years. During the re-emergence of HAdV-41 infections since late 2021, Lineage 2 predominated as previously. Thus, the aetiology of hepatitis cases associated with HAdV-F41 infections during its re-emergence remained obscure and future research on this topic is urgently required.

## Supplementary Material

veac098_Supp

## Data Availability

HAdV-F41 complete genomic sequences generated for this study are available at GenBank accession numbers (ON442312–ON442330, ON532817–ON532827). Accession numbers for pre-existing GenBank sequences used in this study are shown in the phylogenetic trees (Figs 1–3).
